# Nonlinear germanium-silicon photodiode for activation and monitoring in photonic neuromorphic networks

**DOI:** 10.1038/s41467-022-33877-7

**Published:** 2022-10-13

**Authors:** Yang Shi, Junyu Ren, Guanyu Chen, Wei Liu, Chuqi Jin, Xiangyu Guo, Yu Yu, Xinliang Zhang

**Affiliations:** 1 Wuhan National Laboratory for Optoelectronics and School of Optical and Electronic Information, Huazhong University of Science and Technology, 430074 Wuhan, China; 2 Department of Electrical and Computer Engineering, National University of Singapore, 4 Engineering Drive 3, 117583 Singapore, Singapore; 3 Optics Valley Laboratory, 430074 Hubei, China

**Keywords:** Silicon photonics, Optoelectronic devices and components, Integrated optics

## Abstract

Silicon photonics is promising for artificial neural networks computing owing to its superior interconnect bandwidth, low energy consumption and scalable fabrication. However, the lack of silicon-integrated and monitorable optical neurons limits its revolution in large-scale artificial neural networks. Here, we highlight nonlinear germanium-silicon photodiodes to construct on-chip optical neurons and a self-monitored all-optical neural network. With specifically engineered optical-to-optical and optical-to-electrical responses, the proposed neuron merges the all-optical activation and non-intrusive monitoring functions in a compact footprint of 4.3 × 8 μm^2^. Experimentally, a scalable three-layer photonic neural network enables in situ training and learning in object classification and semantic segmentation tasks. The performance of this neuron implemented in a deep-scale neural network is further confirmed via handwriting recognition, achieving a high accuracy of 97.3%. We believe this work will enable future large-scale photonic intelligent processors with more functionalities but simplified architecture.

## Abstract (web summary)

Large-scale silicon-based integrated artificial neural networks lack of silicon-integrated optical neurons. Here, Yu et al, report a self-monitored all-optical neural network enabled by nonlinear germanium-silicon photodiodes, making the photonic neural network more versatile and compact.

## Introduction

Artificial intelligence (AI) has the potential to drastically change our world through accumulating impacts in fundamental science^1,2^, new-type transportation^3,4^, assisted medical treatment^5,6^, etc. Artificial neural network (ANN), a kind of computing architecture inspired by signal processing in the human brain, is one of the major technical pillars for these applications. It contains complex mapping relations in repetitive linear and nonlinear operations. In recent years, however, the required computing capacity for the state-of-the-art ANNs has been doubling every 3.5 months^7^, far overloading Moore’s Law in microelectronics^8^, e.g., electronic computers. Now, silicon (Si) photonics has been recognized as one of the most promising candidates to break through microelectronics bottles owing to its superior interconnect bandwidth, low power consumption and complementary metal-oxide-semiconductor (CMOS) compatibility. According to different implementations, many Si photonic neural network architectures have been proposed to facilitate complex computing tasks, such as diffractive neural networks^9,10^ and optical interference neural networks^11,12^. They utilize diffractive elements or optical interferometers to perform linear operations. The Si photonic interference circuit has been demonstrated as 100× faster than the microelectronic processor but of 1/1000 energy^11^. With the rapidly increasing demand for computational speed and power, Si photonics ANNs provide a promising alternative for AI hardware.

Si photonics neural networks face challenges in large-scale integration due to the lack of proper neurons. Firstly, integrating optical nonlinear material on Si is an open challenge^13,14^. On account of the weak nonlinear effect of Si^15^, heterogeneous integration of other materials is often needed. Although the dye^16,17^, phase-change materials^18,19^ and two-dimensional materials^20,21^ have been proved their optical nonlinearities for all-optical neural networks (AONNs), their stabilities and manufacture abilities are unsatisfactory^22,23^, limiting applications for large-scale networks. For example, two-dimensional materials, such as black phosphorus, are easily irreversible oxidized in air, resulting in poor stability and rapid degradation of the semiconductor properties^24^. Moreover, as the average size of two-dimensional material is limited by the quality of its corresponding three-dimensional precursor, it is hard to produce wafer-scale two-dimensional single crystalline^25^. In addition, the temperature required for crystallization of typical phase-change materials is usually too high for Si-compatible fabrication, hindering the large-scale integration with Si photonics^26^. Secondly, the lack of non-intrusive monitors^27,28^ to prompt the status of the network without interference is another major obstacle. Monitoring and feedback operations enable efficient networks training, node failures detection and environmental fluctuations offset. For a given hardware-based neural network, especially when it is trained completely, such monitors should not change the operating points. However, this is very difficult since a neural network may contain thousands of neurons. For example, the implementation of in situ backpropagation algorithm requires virtually lossless intensity detection in every node^29^. Yet, the conventional light-splitting-and-detection method drifts the operating states and also introduces architecture complexity and accumulated insertion loss.

Here, we propose and demonstrate nonlinear germanium-silicon (Ge-Si) photodiodes (PDs) to construct non-intrusive and self-monitored AONN (SM-AONN) with fully CMOS compatibility. The all-optical power in-power out response is attributed to the intrinsic-absorption-induced free-carrier absorption (FCA) in the Ge thin film. Specially designed electrodes achieve high carrier concentration accumulation via hindering carrier transport. Meanwhile, the Ge-Si heterojunction provides a non-intrusive electrical monitoring signal owing to concomitant photoelectric conversion. In a compact structure of 4.3 × 8 μm^2^ without any optical splitter, the nonlinear activation and monitoring are combined simultaneously, alleviating the issues of complex architecture and operation point drift in conventional ANNs. Experimentally, using the activation and monitoring features, a three-layer SM-AONN enables object classification and semantic segmentation tasks, presenting in situ training and learning with high training accuracy. More layers of SM-AONN can be constructed using optical fiber arrays to connect multiple chips. In addition, the feasibility and performance of this neuron for deep feedforward neural networks are confirmed via the Modified National Institute of Standards and Technology (MNIST) handwriting recognition^30^, achieving a high accuracy of 97.3%.

Our work proves that conventional Group-IV semiconductor technology not only enables all-optical nonlinearity without resorting to other materials but also merges activation and monitoring units. The photonic neural network based on this technology allows for more functionalities, simplified architecture and high accuracy. Due to the material stability and mass-production^31^, we believe that this work will pave a new way toward future high-density integrated photonic intelligent processors.

## Results

### Self-monitored all-optical neural network

**Fig. 1:**
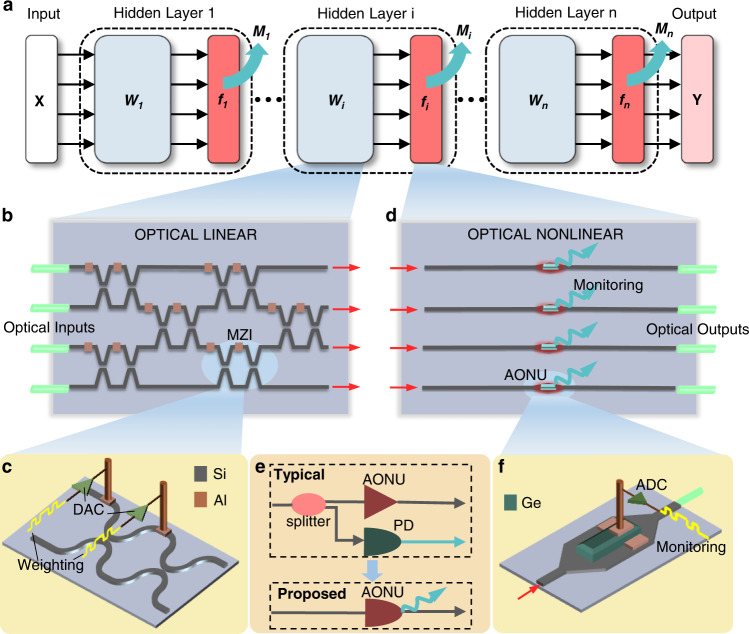
Integrated self-monitored all-optical neuronal circuit. **a** The architecture diagram of the proposed SM-AONN. *X* and *Y* are input and output optical signals in vectors, respectively. *W*_*i*_, *f*_*i*_ and *M*_*i*_ represent linear transformation, nonlinear activation function and electrical monitoring signals for the *i-th* hidden layer, respectively. **b** Reconfigurable Si-based MZI mesh for optical linear operations. Any real matrix, corresponding to any linear transformation, can be decomposed into the product of unitary matrix and diagonal matrix through singular value decomposition. The unitary matrices can be equivalent to MZI networks in triangular or rectangular meshes^56^. The diagonal matrix can be equivalent to MZI arrays. The figure shows the rectangular mesh connected by 6 MZIs, equivalent to any 4 × 4 unitary matrixes. **c** The detailed structure of a tunable Mach-Zehnder interferometer. An MZI consists of two optical phase shifters and two splitters. Any 2 × 2 unitary matrixes can be configured. The MZI network integrated in a photonic chip interconnects with the DAC through wire bonding. Al, aluminum. **d** The Ge-Si all-optical nonlinear block for optical nonlinear activation and electrical monitoring. Four AONUs are included. **e** From light-splitting-and-detection to non-intrusive monitoring. **f** The detailed structure of the AONU. The Ge film coats on the Si waveguide and interacts with light.

Figure 1a shows the architecture of the proposed SM-AONN, consisting of an input layer, multiple hidden layers with monitoring signals and an output layer. In each layer, optical signals are processed by an optical linear transformation and all-optical nonlinear activation building blocks. Being different from the traditional architecture, each nonlinear activation block will produce electrical signals for monitoring the states of each neuron.

Optical linear transformations are implemented using a reconfigurable Si-based Mach-Zehnder interferometer (MZI) mesh, which is an equivalent photonic field programmable gate array, as shown in Fig. 1b. It has been proved that the arbitrary optical linear operations can be carried out by a series of optical beam splitters, phase shifters and attenuators^32,33^, i.e., tunable MZIs^34^. As Fig. 1c shows, voltage signals from the digital-to-analog converters (DACs) are loaded on two thermal-tuning electrodes of the Si-based MZI. The state of each MZI is controlled until the linear operation of the entire network is formed. The weightings between neurons are stored and updated in the voltage information. Note that a complete neuron contains both a linear weighting part and a nonlinear part, and the thermo-optic phase shifter-based linear weighting mesh is indispensable for building complete neurons.

After optical linear operations, the optical signals undergo the Ge-Si all-optical nonlinear units (AONUs) to perform nonlinear processing (activation function), as shown in Fig. 1d. Meanwhile, each AONU provides an electrical monitoring signal to indicate the results of weighting addition and nonlinear operations, by monitoring the input and output optical power of the AONUs. Unlike conventional light-splitting-and-detection solutions, this photoelectric monitoring occurs concomitantly with the optical nonlinear activation in the same structure (Fig. 1e). As shown in Fig. 1f, monitoring signals are drawn from the electrode and converted to the digital domain through the analog-to-digital converters (ADCs). This non-intrusive manner detects the current node states in real-time without changing the network operating point, and thus it enables high performance and stability of the SM-AONN.

### Nonlinear Ge-Si PD-based AONU

**Fig. 2:**
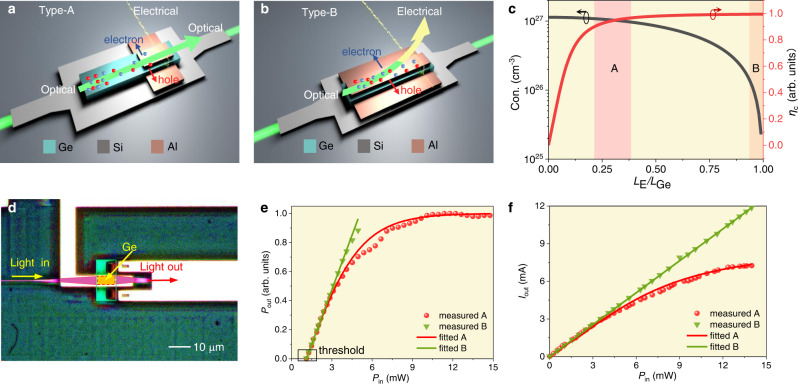
Theoretical and experimental analysis of the Ge-Si AONU. **a** The structure and schematic of the proposed AONU (Type-A). A large number of carriers are accumulated in the non-electrode part of the Ge film, which enhances the nonlinear interaction with light. At the tail end, the carrier movement forms the photocurrent that served as a monitoring signal. The yellow wave ray represents the data flow of the electrical monitoring signals. **b** The structure of the conventional Ge-Si PDs (Type-B). **c** The carrier concentration and collection efficiency versus length ratio. Con., concentration. **d** The false-color image of the AONU. Pink region, Si waveguide. Yellow region, Ge film. Green region, Si slab under Ge film. Red region, metal contacts on Si. The optical signals travel from the Si waveguide into Ge film via evanescent coupling for the desired response. **e** The measured and fitted *P*_out_-*P*_in_ relations. Here, the output optical power is normalized. The actual output optical power of the AONU is between 0 and 1.6 mW under different input optical power, with the optical loss being estimated to be 6.2 dB. The optical loss can be reduced to <3 dB by reducing the optical absorption length or operating at a longer wavelength (with a lower optical intrinsic absorption coefficient). Please see Supplementary Note 5 for more details. **f** The measured and fitted output photocurrents as a function of input optical power.

As a key component of the SM-AONN, the Ge-Si AONU enables all-optical nonlinear activation and non-intrusive monitoring. Figure 2a shows the structure and schematic of it. It is similar to the Ge-Si waveguide PDs applied to photoelectric detection^35,36^ (Fig. 2b). For conventional PDs, the electrodes are with the same length as the Ge film to export out the photo-generated carriers from each part of the absorber. Typically, the output optical power is less concerned. Being different from that, the electrodes herein are omitted where the light is incident to engineer the carrier dynamics. Detailed device geometry and optical field information can be found in Supplementary Note 1. In the electrodeless region (with a small electric field and carrier transit time » carrier lifetime), carriers accumulate and enable the FCA of the Ge film, producing a strong all-optical nonlinear response. In the region with the electrode (with a strong electric field and carrier transit time « carrier lifetime), the carriers are rapidly absorbed by the electrode, and no FCA effect occurs. Fortunately, these collected carriers can be used for optical monitoring. A specific mechanism of the activation function that conforms to the proposed partial electrode structure is given in Supplementary Notes 2 & 3.

By solving the nonlinear Schrödinger equation (NLSE) and carrier rate equation^37,38^ (See Methods), the activation function can be obtained as

1 $${P}_{{{{{{\rm{out}}}}}}}=\frac{\exp (-\alpha {L}_{{{{{{\rm{Ge}}}}}}}){P}_{{{{{{\rm{in}}}}}}}}{1+A[1-\exp (-\alpha ({L}_{{{{{{\rm{Ge}}}}}}}-{L}_{{{{{{\rm{E}}}}}}}))]{P}_{{{{{{\rm{in}}}}}}}}$$

where *A* represents for *στ/2ħωS*. When *A* = 0, the above relationship degenerates into linear absorption. *P*_in_ and *P*_out_ are input and output optical power, respectively, with *α*, *σ*, *τ*, *L*_Ge_*, S* being intrinsic absorption coefficient, absorption cross-section of FCA, carrier lifetime and length of Ge film, as well as incident area. *L*_E_ is the length of the electrode. *ħ* and *ω* represent the reduced Planck constant and optical frequency, respectively. Meanwhile, the concomitant electrical monitoring signal occurs thanks to intrinsic absorption and photoelectric conversion. The FCA effect only transfers momentum between electrons, providing no photocurrent. The nonlinear relationship between the output current and input optical power is expressed as^39^

2 $${I}_{{{{{{\rm{out}}}}}}}=R{P}_{{{{{{\rm{in}}}}}}}\,\tanh (\frac{k{I}_{\max }}{R{P}_{{{{{{\rm{in}}}}}}}})$$

where *I*_out_, *R*, *P*_in_ and *I*_max_ are output current, responsivity at low-power level, input optical power and saturation current, respectively. *k* is a parameter used to change the shape of the curve. Note that this optical monitoring is non-intrusive. The bonding wire is placed ~3 μm above the Si-Ge region, having little influence on the optical signal, and this is the main reason we call it non-intrusive. In addition, the proposed device consumes a portion of optical power to achieve the optical nonlinearity, and the resulting photocurrent is used to realize monitoring at the same time. This is to say, the optical power used to achieve optical nonlinearity is inherently consumed, and no additional optical power is needed to achieve monitoring. This is another important reason we call it non-intrusive.

The length ratio of the electrode to Ge film (*L*_E_*/L*_Ge_) significantly affects the optical-to-optical and optical-to-electrical response. A longer electrode improves the carrier collection efficiency, thereby increasing the output photocurrent^40,41^. However, it reduces the carrier concentration and weaken the FCA effect. The relationship of the carrier collection efficiency and photocurrent can be referred to Supplementary Note 4. Figure 2c shows the carrier concentration and collection efficiency (*η*_c_) versus length ratio. The pink area (*L*_E_*/L*_Ge_ = 0.2–0.4, represented as Type-A) achieves 90% of the maximum value of both. Within this range, a good optical nonlinearity and high optical monitoring responsivity can be obtained simultaneously, and this range can be considered as the optimal ratio. The orange area (*L*_E_*/L*_Ge_ ~ 1) shows the conventional PD (represented as Type-B) with low optical nonlinearity. Figure 2d shows the false-color image of the fabricated AONU. A 4.3 × 8 μm^2^ Ge thin film is epitaxially grown on the Si waveguide. The 3 μm-length electrodes are coated at the optical exportation of Ge. The adopted scheme (Type-A) corresponds to *L*_E_*/L*_Ge_ of 0.375. See Methods for more fabrication details.

Here, we experimentally verified the optical and electrical responses of the proposed AONU, compared with a reference conventional PD. The *P*_out_-*P*_in_ relations are shown in Fig. 2e. For Type-A, the output power is linear at low input, and then gradually flattens as the power increases, showing obvious *P*_out_-*P*_in_ nonlinearity. However, the curve of Type-B is linearly tangent to that of Type-A. At the same input, the difference between the two curves contributes to the FCA. The threshold of the nonlinear activation is about 1.1 mW. Such a low threshold requirement is very beneficial for low power consumption and for driving the nonlinearity units of next level. The activation functions are fitted by Eq. (1), as the solid line shown in Fig. 2e. On the other hand, the measured output photocurrents are shown in Fig. 2f. Although the linearity is slightly reduced, the photocurrent still increases monotonously with the input optical power, so that the input optical power can be uniquely determined and monitored from the output current. Combined with the *P*_in_*-P*_out_ relation, the output optical power can also be determined. The photodetection metrics including the responsivity, bandwidth and dark current can be referred in Supplementary Note 6. The bandwidth is influenced by the doping of the AONU and the detailed analysis is given in Supplementary Note 7.

### Large scale SM-AONN performance

**Fig. 3:**
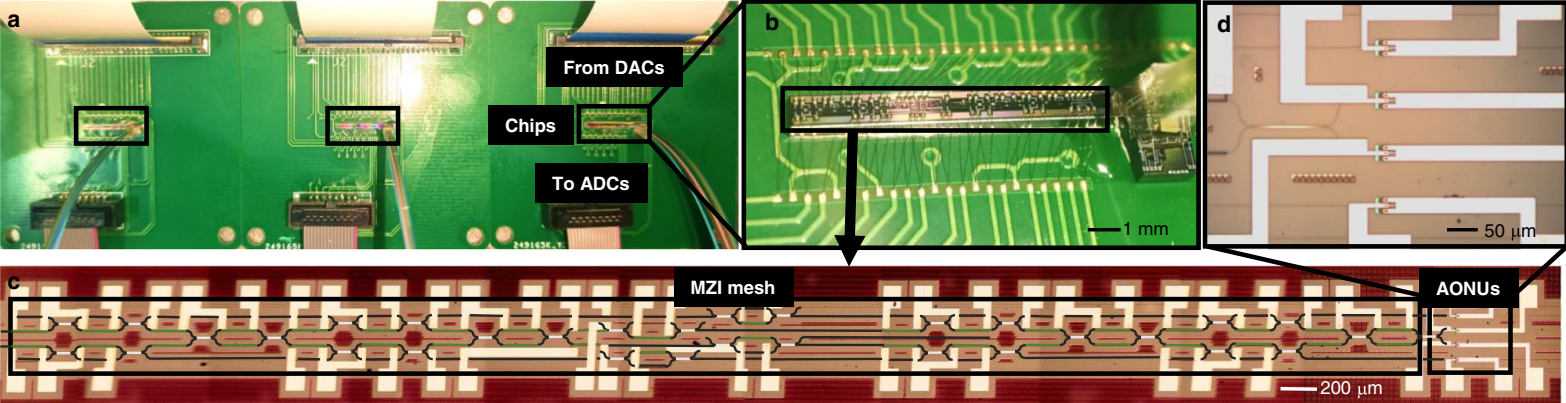
Three-layer SM-AONN. **a** The photo of the connected three-layer networks. **b** The packaged single layer within one chip. **c** The false-color image of MZI mesh and AONUs. Black, Si waveguide. Green, electrodes of thermally-tunable MZI. **d** The detailed AONUs of a single layer. The structure of each AONU has been shown in Fig. 2d.

Having proved that the state of each neuron can be obtained from the monitoring signals, the performance of the entire neural network is characterized. We prepare a scalable three-layer fully connected feedforward neural network using MZI mesh and the proposed AONUs, as shown in Fig. 3a. Although the three-layer network can be built on one chip with the same fabrication process, we split it into three chips and connect them using optical fiber arrays, for easy comparison and arbitrary combination. More importantly, more layers of networks can be constructed using optical fiber arrays to connect multiple chips. Here, three layers are sufficient to demonstrate the following machine learning tasks with high accuracy. Figure 3b shows one layer of the packaged SM-AONNs, consisting of four neurons with 16 MZIs and four nonlinear units. The MZI mesh and nonlinear units are present in Fig. 3c, d, respectively.

**Fig. 4:**
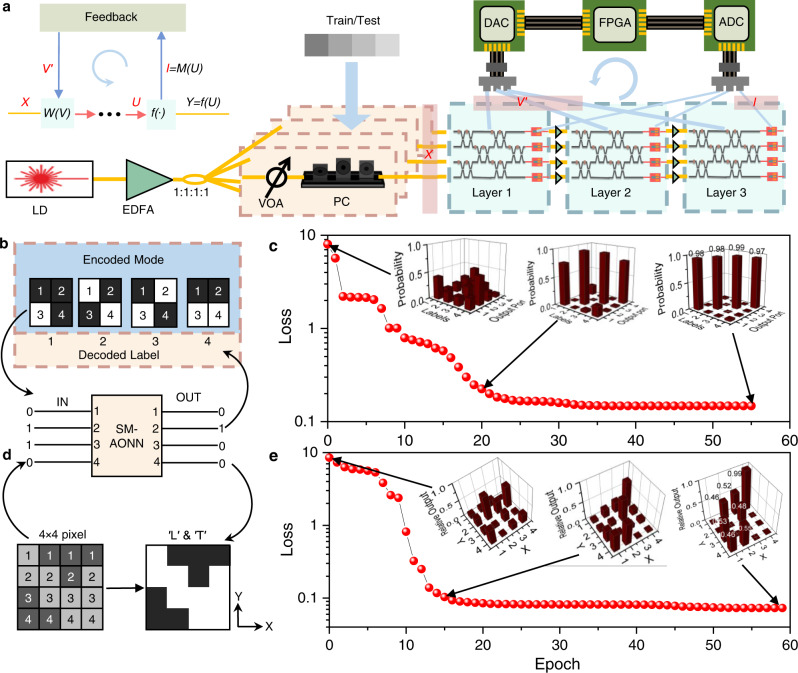
Training and results of three-layer neural networks. **a** The set-up diagram of training. The panel on the left side represents the data flow during training, where *W(V)*, f (∙) are linear and nonlinear operations, respectively. *X*, *Y* are the input of the first layer and the output of the last layer, respectively. *U* and *I* are the optical input and electrical output of the last layer of AONU, respectively. *V* is the voltage that controls the weighting. LD, Laser. EDFA, Erbium-Doped Fiber Amplifier. VOA, Variable Optical Attenuator. PC, Polarization Controller. **b** Introduction of the classification task. Four modes are utilized for simplified classification tasks. The classified patterns are represented by 4 × 1 vectors. Black pixel is represented by ′1′ and input, while white is ′0′ and no input. The task is to train the neural network so that all modes are output only at their labeled ports. **c** The results of the classification task. The insets are the probabilities of each mode output from the four ports. **d** Introduction of the semantic segmentation task. The input in the dark gray area is set to 0.9 and the output is 1. The input in the light gray area is set to 0.1 and the output is 0. **e** The results of the semantic segmentation task. The insets are the relative outputs of 16 pixels.

The basic operations of neural networks are training and inference. Compared with inference, training consumes most of the computing power in neural networks. However, it can be completed quickly and automatically, using self-monitoring electrical signals combined with special processing chips and optoelectronic integration. The training set of machine learning tasks consists of a series of vectors of inputs and outputs, being encoded on optical power. As shown in Fig. 4a, the input optical signals are processed by the photonic chip to obtain the real optical outputs. Being different from the conventional training method, the real output is read by monitoring signals rather than external PDs. A loss function such as cross-entropy^42^ is defined to evaluate the distance between the real outputs and training-set predicted outputs. The difference is eliminated with iteration by feedback algorithms such as backpropagation^43^ in special processing chips. Then, the SM-AONN is trained completely. The detailed in situ training implementation can refer to Supplementary Note 8.

Experimentally, the simplified object classification and semantic segmentation tasks are performed. As shown in Fig. 4b, we utilize two-valued optical intensities to encode the labels of four input targets, for example, ′0110′ for input and ′0100′ for output are represented for ′target 2′. At the optical input port, only ports 2 and 3 are configured to pass through via the variable optical attenuators (VOAs). When the neural network is successfully trained, only port 2 is expected to be the optical output. In real application, the targets can represent different grayscale images. Figure 4c shows the relationship of the loss function and iterations. The output histograms of the initial state, the intermediate state of the 20 iterations and the final state are shown as the insets. In the initial state, the output of each mode is chaotic, since the weightings of the MZI network are given randomly. With the reconstruction of weightings, the recognition of each mode becomes clearer. Being fully configured, the output probability of each mode at the correct port exceeds 97%. Similarly, the training for semantic segmentation is present. As a 4 × 4-pixel image shown in Fig. 4d, the gray levels of the ′L′ and ′T′-type regions are greater than others. After training, the gray levels of ′1′ and ′0′ are contrastive to identify ′L′ and ′T′ in the image. Since each input to SM-AONN is a column vector (in the Y direction), the sum of normalized output power in the Y direction remains unity. As Fig. 3e shows, when the number of iterations exceeds only 15 epochs, the output of each port is near the expectation of 50% for two input ports and 100% for one input port. For these two experiments, the error analysis can refer to Methods. The successful training of two different tasks has demonstrated the general configuration task and the powerful learning ability of the SM-AONN. Thanks to the electrical monitoring signals, the training results have extremely high expected accuracy. Large-scale training tasks are fully automated with the help of electronics.

Here, we use the digital computing as an example. Actually, the demonstrated photonic neuromorphic computing architecture is analog in nature and can be used for analog computing as well. This is because the MZI weighting network can directly handle the multiplication of complex-valued data, and the optical nonlinear response is also a continuous-valued input-output function. The difference between analog computing and digital computing is only the form of the input and output data sets. If the current digital input of ′0′ or ′1′ is replaced with a continuous-time optical intensity, analog computing can be performed.

**Fig. 5:**
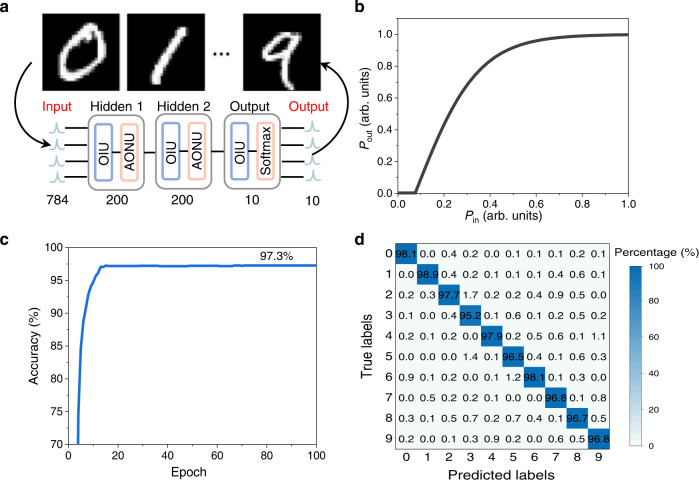
Handwriting recognition with a deep feedforward AONN. **a** The 10 digits and the structure of the neural nwtwork. OIU, optical linear unit, is a series of weightings between neurons in the software. **b** The activation functions normalized by a maximal input power of 15 mW. **c** The accuracy result obtained by the test. **d** The tested confusion matrix.

Going forward, we introduce the obtained nonlinear optical responses as nonlinear activation functions in a three-layer deep feedforward neural network for the MNIST handwriting recognition, to further test large-scale data processing capability. The MNIST data set consists of 60,000 784-pixel images, therein 50,000 and 10,000 images are used for training and testing, respectively. These images contain handwritten digits from 0 to 9, as shown in Fig. 5a. The deep feedforward neural network consists of two hidden layers containing 200 neurons and an output layer containing 10 neurons. The input is a 784 × 1 vector, and the output is a 10 × 1 vector. The output layer adopts the *Softmax* activation function to convert the output results into probability. The proposed Ge-Si AONU is extracted as the activation function for the hidden layers. The activation function with normalized input and output is shown in Fig. 5b. The simulation utilizes the conjugate gradient backpropagation algorithm to iterate 100 times, and the loss function is cross-entropy. An accuracy of 97.3% and corresponding confusion matrix are shown in Fig. 5c and d, respectively. Each column of the matrix represents the instances in a predicted label, while each row represents the instances in a true label. The diagonal elements represent the probabilities that are correctly predicted. These results show that our nonlinear unit has high performance on representative machine learning tasks.

## Discussion

One of the key advantages of the AONU is the ability to non-intrusively observe the optical energy. The experimental and emulational comparisons on the performance and stability are provided in Supplementary Note 9. Indeed, the results indicate a more stable and better performance for the proposed “non-intrusive” scheme. Compared to the intrusive monitoring with different degrees of perturbation, the non-intrusive scheme shows a smoother activation function and improved accuracies of 1.7–4% in handwritten recognition. Furthermore, the iterations to reach the maximum accuracy is much less, resulting in a decreased training cost. In addition, when the neural network is trained completely, the accuracy fluctuation is much smaller, which means a better stability on inferring tasks. On the other hand, photonic neural networks are large-scale and dynamically tunable circuits, and their control becomes enormously difficult due to manufacturing variations and thermal crosstalk^44^. Fortunately, the non-intrusive monitoring provides a calibration capability by compensating the fabrication errors and environmental fluctuations. In the training process, the monitoring enables non-intrusive intensity detection of each node, to implement in situ gradient measurements and forward or backpropagation algorithms^29^. This method can enable highly efficient gradient calculation in training. When an already trained neural network is working, the non-intrusive monitoring feature can obtain information about environmental fluctuations without changing the operating point of the network^27^. On this basis, the network can be dynamically tuned and calibrated without introducing other disturbances.

Another main advantage of the photonic neural network is potentially possessing higher speed and energy efficiency compared to electronics^10,45^. Typically, the computing speed is defined as the number of operations per second (FLOPS). For our demonstrated system, the FLOPS is calculated to be 1.92 × 10^12^ operations per second with a 20 GHz detection bandwidth. In principle, such a computing speed is one order of magnitude faster than electronic neural networks which are usually restricted to a GHz clock rate^46^. The consumed energy is calculated to be ~0.27 pJ per operation in our system, better than an “ideal” electronic computer (1 pJ per operation, assuming no energy is used on data movement) and two orders of magnitude better than conventional graphics processing units (GPUs) (100 pJ per operation)^47^. Please see Supplementary Note 10 for the detailed calculation and comparison. On the other hand, in the photonics system, the energy required for the optical nonlinearity of the Si-Ge system is relatively higher than that of some other materials^48^, but it has the advantages of CMOS fabrication compatibility and compact structure that other material systems may not have.

The scalability of the photonic neural network is an important challenge. Typically, some form of nonlinearity is required to implement the thresholding effect of a neuron in the neural networks. However, optical nonlinear responses are comparatively power inefficient, and the neuron output is often weaker than its input^14^. Thus, previous works utilized optical amplifiers^49,50^, optical-electrical-optical conversion^51^ or all-optical carrier regeneration^18^ to alleviate this issue. These methods also bring additional optical and electrical power consumption. By contrast, an advantage of our scheme is that only the loss of the optical nonlinear part needs to be considered, while the loss from optical splitters and monitoring is avoided. This might be competitive as the neural network scales up. At present, we use off-chip EDFAs to pump the network. Recently, Liu, et al.^52^ achieved on-chip erbium-doped waveguide amplifiers with a gain up to 30 dB. This would be suitable to simultaneously address the challenges of multi-layer scaling and on-chip integration.

Aiming at solving the issues of large-scale Si-based integrated ANNs, we have demonstrated that the specifically designed nonlinear Ge-Si PD enables both all-optical activation and non-intrusive monitoring. The SM-AONN based on this technology achieves 97.3% accuracy on open machine learning tasks. The advantages of the Ge-Si PD-based SM-AONN include: (1) Material advantages. Ge is a kind of material with stability and CMOS compatibility. (2) All-optical operations. The photoelectric conversion only occurs during training. There is no need for the information exchange between optical and electrical domains once trained. (3) Non-intrusive monitoring. The network supports automatic training, node failures analysis and environmental fluctuations monitoring without disturbing the operation points. (4) Simplified architecture. The activation and monitoring units are merged in the same device with compact footprint. (5) Large scale. Multiple layers of SM-AONN can be constructed using optical fiber arrays to connect multiple chips. (6) High accuracy. A deep neural network utilizing this new activation function shows high performance. In addition, due to characteristics of the Si MZI network and Ge nonlinearity, this network may also draw interests in quantum networks^53,54^ or mid-infrared applications^55^. We believe that this work is promising for future large-scale optical intelligent neuromorphic systems.

## Methods

### Analysis coupled equations

The interaction process of intrinsic absorption and FCA can be described by the nonlinear NLSE equation

3 $$\frac{{{{{{\rm{d}}}}}}I}{{{{{{\rm{d}}}}}}z}=-\alpha I-\beta {I}^{2}-\sigma NI$$

and the carrier rate equation

4 $$\frac{\partial N}{\partial t}=\frac{\alpha }{\hslash \omega }I+\frac{\beta }{2\hslash \omega }{I}^{2}-\frac{N}{\tau }$$

where *I* and *N* are optical intensity and carrier concentration, respectively, with *α*, *β*, *σ* and *τ* being intrinsic absorption coefficient, two-photon coefficient, absorption cross-section of FCA and carrier lifetime of the Ge. Here, *β* = 0. *ħ* and *ω* represent the reduced Planck constant and optical angular frequency, respectively. *z* is the light propagation direction and *t* is the time.

### Device fabrication

The device is fabricated using a silicon-on-insulator wafer with 220 nm thick Si top layer and 2 µm buried oxide. The Si layer is etched into strip waveguides for the pattern of the MZIs and Si slab under Ge film. Then, the Si top layer is implanted using different doses of boron ions to form the P-type regions. A 500 nm-thick Ge film is grown on the P-type doped Si slab. On the top of Ge film, phosphorus ions are implanted with ~100 nm-depth to form the N-type region of a PIN junction. The titanium nitride (TiN) heater of 120 nm in thickness is deposited 2 μm above the Si waveguide for thermal tuning. Finally, metal electrodes are fabricated and connect to Si, Ge and TiN through via holes.

### Error analysis

The training of the neural network relies on the monitoring photocurrent of the AONU, and then the weighting values are loaded on the thermally tuned MZI network in the form of voltages. The photodetector noise (*σ*_D_) and the voltage fluctuation applied on MZIs (*σ*_Φ_) are the dominant error sources. In the experiments, we used DACs with 10-bit precision and a three-layer 4 × 4 matrix with *σ*_Φ_ estimated to be 10^−3^, as well as a photodetector noise of *σ*_D_ = 1.8 × 10^−3^ under a mean photocurrent of ~1 mA. We carried out the following steps to numerically simulate the performance with the *σ*_D_ and *σ*_Φ_. For the trained 4 × 4 unitary matrices *U*, we calculate a set {*V*_MZI_} that encodes the matrix. We assume phase-encoding errors *δV*_MZI_ is a random variable sampled from a Gaussian distribution G(0, *σ*_Φ_). We obtain a new set of perturbed phases {*V*_MZI_ + *δV*_MZI_} and perturbed 4 × 4 unitary matrices *U*′. During forward propagation, every time a matrix multiplication is performed for a result **v** = *U*′ *·* **u** (**u** is input vector), we add a set of random photodetection errors *δ***v** as the perturbed output vector **v**′ *=*  **v** + *δ***v**, where we assume each *δ***v** is a random variable sampled from a Gaussian distribution G(0, *σ*_D_*·|***v** | ). Then perturbed optical output is derived from **v**′ and the accuracy is calculated. Repeating 50 times, the final accuracy is estimated to be ~98%. We attribute other errors to the fabrication error and thermal crosstalk of the linear networks. The fabrication error can be compensated by pre-calibration steps, while the thermal crosstalk can be reduced by adding thermal isolation trenches.

## Supplementary information

Supplementary Information

## Data Availability

All the data supporting this study are available in the paper and Supplementary Information. Additional data related to this paper are available from the corresponding authors upon request.

## Code availability

The simulation and computational codes for this study are available from the corresponding authors on reasonable request.
