# Supplementary material for: Nonlinear germanium-silicon photodiode for activation and monitoring in photonic neuromorphic networks
Source: Nat Commun. 2022 Oct 13;13:6048. doi: 10.1038/s41467-022-33877-7 (PMC9561110; doi:10.1038/s41467-022-33877-7)
Supplement: Supplementary file 1 — Supplementary Information [file 41467_2022_33877_MOESM1_ESM.pdf]

Supplementary Information for

Nonlinear germanium-silicon photodiode for activation and monitoring in photonic neuromorphic networks

Yang Shi[1], Junyu Ren[1], Guanyu Chen[1,2], Wei Liu[1], Chuqi Jin[1], Xiangyu Guo[1], Yu Yu[1,3*] & Xinliang Zhang[1,3]

[1]*Wuhan National Laboratory for Optoelectronics and School of Optical and Electronic Information, Huazhong University of Science and Technology, Wuhan, 430074, China*

[2]*Department of Electrical and Computer Engineering, National University of Singapore, 4 Engineering Drive 3, 117583, Singapore*

[3]*Optics Valley Laboratory, Hubei, 430074, China*

**e-mail: yuyu@mail.hust.edu.cn*

Table of Contents

Ⅰ Device structure

Supplementary Note 1. **Device schematic and simulation**

Ⅱ Operation principle

Supplementary Note 2. **Analytical coupled equation**
Supplementary Note 3. **Activation function**
Supplementary Note 4. **Monitoring photocurrent**

Ⅲ Device characterization

Supplementary Note 5. **Measured optical response**
Supplementary Note 6. **Photodetection performance**
Supplementary Note 7. **Influence of doping**

Ⅳ Neuromorphic photonic processor

Supplementary Note 8. **Training SM-AONN**
Supplementary Note 9. **Non-intrusive/intrusive monitoring**
Supplementary Note 10. **Computing speed and energy**

I Device structure

Supplementary Note 1. **Device schematic and simulation**

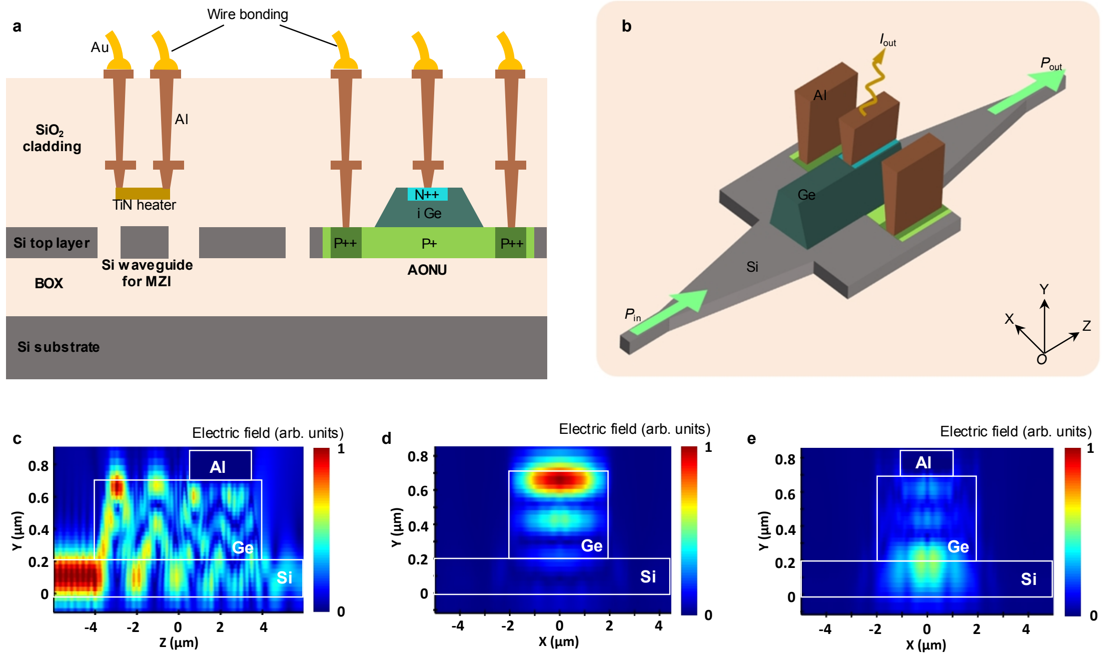

Supplementary Figure 1. Device and optical simulation. a The cross-section of the thermal-tuning Si waveguide and the AONU on the SOI platform. Au, gold. **b** The three-dimensional structure of the AONU. P_{in}, optical input. P_{out}, optical output. I_{out}, electrical output. **c** The simulated optical field along the light propagation direction. **d** The optical field of the cross-section of the electrodeless region (z= -3 μm). **e** The optical field of the cross-sections of the electrode region (z= 2 μm).

The self-monitoring all-optical neural network (SM-AONN) is fabricated on the silicon-on-insulator (SOI) platform. From top to bottom, it contains a 3-μm-thick silica (SiO_2) cladding layer, a 220-nm-thick top silicon (Si) layer, a 2-μm-thick buried dioxide (BOX) layer and a 750-μm-thick Si substrate. The Mach-Zehnder interferometer (MZI) weighting network consists of Si waveguides and titanium nitride (TiN) heaters suspended above. The all-optical nonlinear unit (AONU) consists of waveguides on Si top layer, upper germanium (Ge) film and aluminum (Al) electrodes. They connect with the electrical circuit via wire bonding. We present the cross-section of the thermal-tuning MZI and the AONU in Supplementary Fig. 1a. The Si waveguide is 500×220 nm^2 in cross-section dimension, being designed for single-mode transmission at 1550 nm. The AONU is based on an n-i-p junction consisting of N++ Ge, intrinsic Ge (i-Ge) and P+ Si. The heavily doped N++ Ge and P++ Si enable good ohmic contact with the metals.

The overall structure of the AONU is shown in Supplementary Fig. 1b. We place the AONU on a Si multimode interference (MMI) structure, consisting of two 500×220 nm^2 single-mode waveguides at the edges as input/output and a 9-μm-length multimode waveguide in the middle. They are connected through 40-μm-length tapers, which enable the low-loss adiabatic optical mode evolution. The Ge film is 4.3 μm wide, 8 μm long and 0.5 μm thick. The partial electrode structure is adopted. The doping region and metal contacts are 3-μm in length.

Using the device geometry described above, we simulate the optical field distributions using 3D FDTD method, as shown in Supplementary Figs. 1c-e. Supplementary Fig. 1c shows the simulated

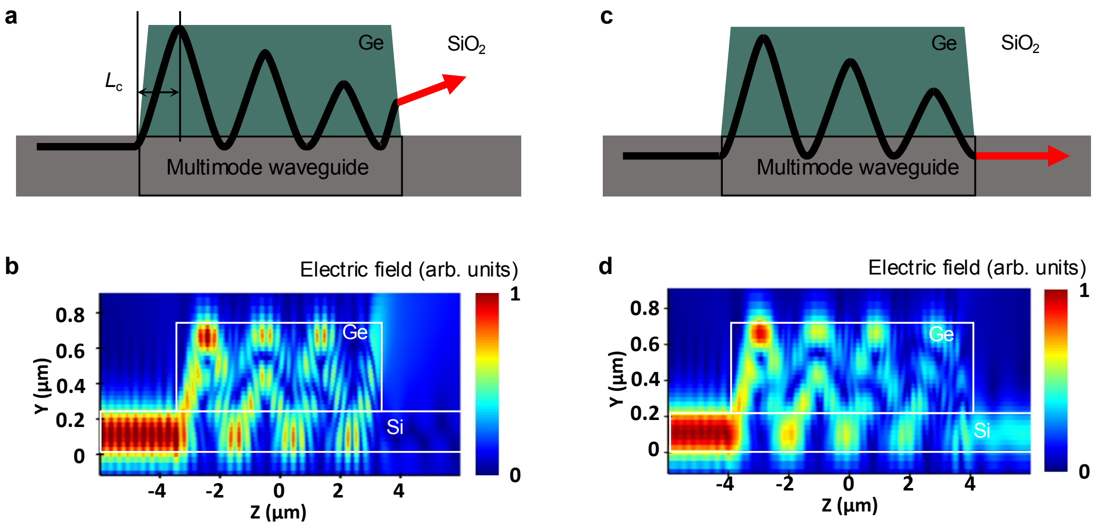

Supplementary Figure 2. Optical field distribution with different contact lengths. a The schematic of the AONU with a contact length of 7 μm, in which the remaining light scatters into the silica. L_C, coupling length. **b** The simulated optical field distribution of the structure with 7 μm contact length. **c** The schematic of the AONU with a contact length of 8 μm, in which the remaining light distributes in the Si output waveguide. **d** The simulated optical field distribution of the structure with 8 μm contact length.

optical field along the light propagation direction (z-axis). The optical field is evanescently coupled between Si multimode waveguide and Ge film periodically, with a coupling length of ~ 1 μm. Meanwhile, the optical intensity gradually weakens due to the absorption of Ge. Supplementary Figs. 1d and 1e show the optical field of the electrodeless region (z = -3 μm) and the electrode region (z = 2 μm), respectively.

The contact length of the multimode waveguide and the Ge film determines the output position of light. When the contact length is an odd multiple of the coupling length, the optical power at the end of the Ge region is scattered into the SiO_2 (Supplementary Figs. 2a and 2b. The contact length is 7 μm), resulting in a large loss. When the contact length is an even multiple of the coupling length, the output optical power is primarily distributed in the Si output waveguide (Supplementary Figs. 2c and 2d. The contact length is 8 μm), resulting in little loss. By elaborately designing the parameters, the scattering loss is as low as 0.22 dB.

Ⅱ Operation principle

Supplementary Note 2. **Analytical coupled equation**

Assuming that the Ge absorber is one-dimensional and there is no electric field, the interaction process of intrinsic absorption, the two-photon absorption and the free-carrier absorption (FCA) can be described by the nonlinear Schrödinger equation (Supplementary Equation (1)) and the carrier rate equation (Supplementary Equation (2)):

$$\frac{\mathrm{d}I(z)}{\mathrm{d}z} = -\alpha I(z) - \beta I^2(z) - \sigma N(z,t) I(z) \tag{1}$$

$$\frac{\partial N(z,t)}{\partial t} = \frac{\alpha}{\hbar\omega} I(z) + \frac{\beta}{2\hbar\omega} I^2(z) - \frac{N(z,t)}{\tau} \tag{2}$$

where $I(z)$ and $N(z, t)$ are optical intensity and carrier concentration, respectively, with α, β, σ and τ being intrinsic absorption coefficient, two-photon coefficient, free-carrier cross-section and carrier

lifetime of Ge. Here, β=0 when the device works at the wavelength of 1550 nm. $\hbar$ and ω represent the reduced Planck constant and optical angular frequency, respectively. The condition for the steady-state solution is:

$$\frac{\partial N(z,t)}{\partial t} = 0 \tag{3}$$

Solves Supplementary Equations (2-3) for z to obtain:

$$N(z) = \frac{\tau\alpha}{\hbar\omega} I(z) \tag{4}$$

Substituting Supplementary Equation (4) into (1) to obtain:

$$\frac{\mathrm{d}I(z)}{\mathrm{d}z} = -\alpha I(z) - \frac{\sigma\tau\alpha}{\hbar\omega} I^2(z) \tag{5}$$

Given boundary condition:

$$\begin{aligned} I(0) &= I_{\mathrm{in}} \\ I(z) &= I_{\mathrm{out}} \end{aligned} \tag{6}$$

Solves Supplementary Equations (5-6) to obtain:

$$I_{\mathrm{out}}(z) = \frac{e^{-\alpha z} I_{\mathrm{in}}}{1 + \frac{\sigma\tau}{\hbar\omega}(1 - e^{-\alpha z}) I_{\mathrm{in}}} \tag{7}$$

According to the definition formula of optical intensity $I=P/S$ (where P is the optical power and S is the incident area), the solution can be finally expressed as:

$$P_{\mathrm{out}}(z) = \frac{e^{-\alpha z} P_{\mathrm{in}}}{1 + \frac{\sigma\tau}{\hbar\omega S}(1 - e^{-\alpha z}) P_{\mathrm{in}}} \tag{8}$$

Supplementary Note 3. **Activation function**

In Supplementary Fig. 3a, we show the partial electrode structure that the proposed device used. The device consumes a portion of optical power to generate the carriers through the intrinsic absorption in Ge. A key point is whether the carriers can transit to the electrode within the carrier lifetime τ_s (the time for carrier recombination). This determines whether or not the carriers gather and produce the FCA effect. In the electrodeless region (with a weak electric field and carrier transit time $\tau_t >> \tau_s$), carriers accumulate and enable the FCA effect. In the region with the electrode (with a strong electric field and $\tau_t << \tau_s$), the carriers are rapidly collected by the electrode, and no FCA effect occurs. Fortunately, these collected carriers can be used for optical monitoring. For the conventional photodiode (Supplementary Fig. 3b), the electric field is distributed in the entire Ge region, and it will facilitate the transport of carriers, thereby suppressing the carrier accumulation and optical nonlinearity.

Along the light propagation direction (z-axis) of the partial electrode structure, the electric field is:

$$E(z) = \begin{cases} 0 & 0 \le z \le L_{\mathrm{Ge}} - L_{\mathrm{E}} \\ V_{\mathrm{b}} / h_{\mathrm{Ge}} & L_{\mathrm{Ge}} - L_{\mathrm{E}} < z \le L_{\mathrm{Ge}} \end{cases} \tag{9}$$

where V_b is the built-in voltage of the photodiode, and h_{Ge}, L_{Ge} and L_E are the Ge height, Ge length and

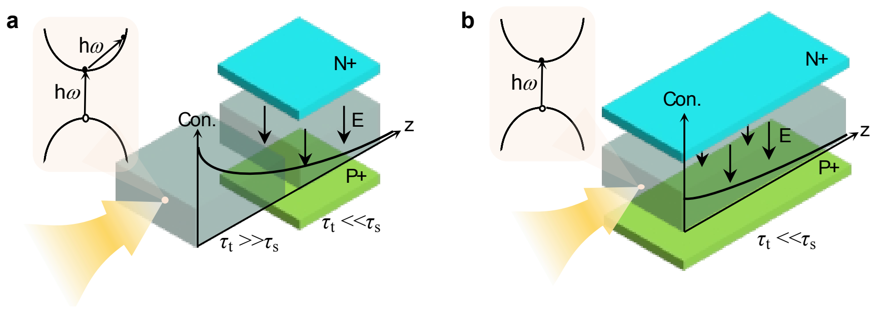

Supplementary Figure 3. Mechanism of optical-to-optical response. a The schematic of the proposed AONU with a partial electrode structure. **b** The schematic of the conventional photodiode. Con., carrier concentration. Con., carrier concentration. E, electric field. N+, N-doped Ge. P+, P-doped Si.

electrode length, respectively. In the absence of external bias, the electric field is derived from the built-in voltage that can be expressed as:

$$V_b = \frac{kT}{q}\ln\left(\frac{N_D N_A}{n_i^2}\right) \tag{10}$$

where N_D, N_A and n_i are the doping concentrations of the N++ Ge, P+ Si and i-Ge region, respectively. They determine the built-in electric field and subsequently engineer the carrier transport. k, T and q are Boltzmann constant, temperature and electron charge, respectively.

The carrier transit time can be expressed as:

$$\tau_t = \begin{cases} \infty & 0 \le z \le L_{Ge} - L_E \\ \dfrac{h_{Ge}}{\mu E(z)} & L_{Ge} - L_E < z \le L_{Ge} \end{cases} \tag{11}$$

Here, τ_t=50 fs<<τ_s (~1 ns) in the electrode region (E=10 kV cm^{-1}, h_{Ge}=0.5 μm, μ=3900 cm^{2}V^{-1}s^{-1} for calculation). According to Supplementary Equation (4), the final carrier concentration distribution is expressed as:

$$N(z) = \begin{cases} \dfrac{\dfrac{\tau\alpha}{\hbar\omega}P_{in}e^{-\alpha z}}{1+\dfrac{\sigma\tau}{\hbar\omega}P_{in}(1-e^{-\alpha z})} & 0 \le z \le L_{Ge} - L_E \\ 0 & L_{Ge} - L_E < z \le L_{Ge} \end{cases} \tag{12}$$

Therefore, in the electrodeless region, carriers accumulate and enable the optical nonlinear effect. In the region with the electrode, the carriers are rapidly collected by the electrode, and only the intrinsic absorption occurs. The strength of the nonlinearity can be evaluated by the average carrier concentration. Finally, for the proposed partly electrode structure, the nonlinear activation function can be extracted from (according to Supplementary Equation (8)):

$$P_{out} = \frac{e^{-\alpha(L_{Ge}-L_E)}P_{in}}{1+\dfrac{\sigma\tau}{\hbar\omega S}[1-e^{-\alpha(L_{Ge}-L_E)}]P_{in}} \cdot e^{-\alpha L_E} = \frac{e^{-\alpha L_{Ge}}P_{in}}{1+\dfrac{\sigma\tau}{\hbar\omega S}[1-e^{-\alpha(L_{Ge}-L_E)}]P_{in}} \tag{13}$$

Supplementary Note 4. **Monitoring photocurrent**

The photocurrent originates only from the intrinsic absorption, and the FCA does not contribute to it. Under low optical power incidence, the photocurrent is proportional to the input optical power, expressed as:

$$I_{\text{out}} = \frac{q\lambda}{2\pi\hbar c}\eta_{\text{cou}}\eta_{\text{c}}(1-e^{-\alpha L_{\text{Ge}}})P_{\text{in}} \tag{14}$$

where η_{cou} and η_{c} are the optical coupling efficiency from the Si waveguide to the Ge film and the carrier collection efficiency, respectively. λ and c are the wavelength and the speed of light, respectively. Among these factors, η_{c} is quite different with/without electrodes. According to supplementary reference [1], for E=0 and 10 kV cm^{-1}, it is:

$$\eta_{\text{c}} = \begin{cases} 0.5 & 0 \le z \le L_{\text{Ge}} - L_{\text{E}} \\ 0.9 & L_{\text{Ge}} - L_{\text{E}} < z \le L_{\text{Ge}} \end{cases} \tag{15}$$

Then, the photocurrent of our device can be expressed as:

$$I_{\text{out}} = \frac{q\lambda}{2\pi\hbar c}\eta_{\text{cou}}[0.5\cdot(1-e^{-\alpha(L_{\text{Ge}}-L_{\text{E}})})+0.9\cdot e^{-\alpha(L_{\text{Ge}}-L_{\text{E}})}(1-e^{-\alpha L_{\text{E}}})]P_{\text{in}} \tag{16}$$

The average carrier collection efficiency can be used to evaluate the optical-electrical response and it is defined as:

$$\eta_{\text{c, ave}} = \frac{0.5\cdot(1-e^{-\alpha(L_{\text{Ge}}-L_{\text{E}})})+0.9\cdot e^{-\alpha(L_{\text{Ge}}-L_{\text{E}})}(1-e^{-\alpha L_{\text{E}}})}{1-e^{-\alpha L_{\text{Ge}}}} \tag{17}$$

When the input optical power is large enough, the photocurrent gradually tends to saturate due to the space charge screening effect [2]. At this point, the photocurrent can be expressed as:

$$I_{\text{out}} = RP_{\text{in}}\tanh\left(\frac{kI_{\max}}{RP_{\text{in}}}\right) \tag{18}$$

where R and $I_{\max}$ are responsivity at low-power level and saturation current, respectively. k is a parameter used to change the shape of the curve.

Ⅲ Device characterization

Supplementary Note 5. **Measured optical response**

Supplementary Fig. 4a shows the output optical power versus input optical power of the AONU. The output optical power is between 0-1.6 mW under different inputs, with the optical loss being estimated to be 6.2 dB. The optical loss of the device is determined comprehensively by the absorption coefficient and Ge length. For a given material, the absorption coefficient is related to the wavelength. We simulate the optical loss with different Ge length at 1550 and 1700 nm, as shown in Supplementary Fig. 4b. At 1550 nm, the simulated loss is 5.7 dB (using the 8-μm Ge length in our device), and it accords well with the experimental result. Obviously, the optical loss can be further decreased by increasing the operating wavelength or reducing Ge length. For example, with an 8-μm device operating at 1700 nm or a 4-μm device operating at 1550 nm, the optical loss will be reduced to be 3 dB. Reasonably, the responsivity of monitoring will be reduced to some extent.

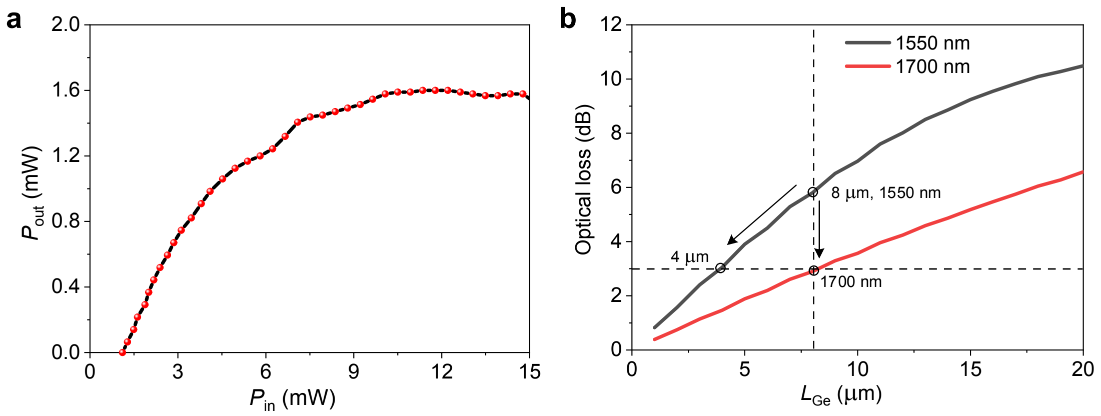

Supplementary Figure 4. Optical response and loss. a The measured output optical power versus input optical power of the AONU. **b** The simulated optical loss with different Ge length at 1550 and 1700 nm.

Supplementary Note 6. **Photodetection performance**

In this section, we present the optical detecting metrics of the AONU, including dark current, responsivity and bandwidth. The current-voltage (I-V) characteristics of the device are measured using a source meter, a probe station and a tunable laser. The results are shown in Supplementary Fig. 5a. The dark current is as low as 0.013 nA at 0 V and 1.1 nA at -1 V, respectively. This is beneficial from the compact footprint of our device and the good fabrication process. The photocurrent is measured at 1550 nm under a received optical power of -5.0 dBm. The measured responsivity is 0.83 A/W. The photocurrent is almost constant under 0 to -4 V owing to the strong built-in electric field that is capable of sweeping out most of the photo-generated carriers within the lifetime.

Small-signal radio-frequency measurements are performed using a vector network analyzer in the test range of 10 MHz to 70 GHz with 50 Ω load resistance. The normalized S_{21} parameters are shown in Supplementary Fig. 5b. The measured 3-dB bandwidth is 20.2 GHz. We analyze the result using the equivalent circuit model presented in Supplementary Fig. 5c. The detailed meaning of each element can be referred to supplementary reference [3]. The extracted series resistance (R_s) and capacitance of the bonding wires (C_p) are 62.1 Ω and 15 fF, respectively. Without using the bonding wires, the calculated bandwidth of the photodiode itself is 51 GHz, as the red line shown in Supplementary Fig. 5b.

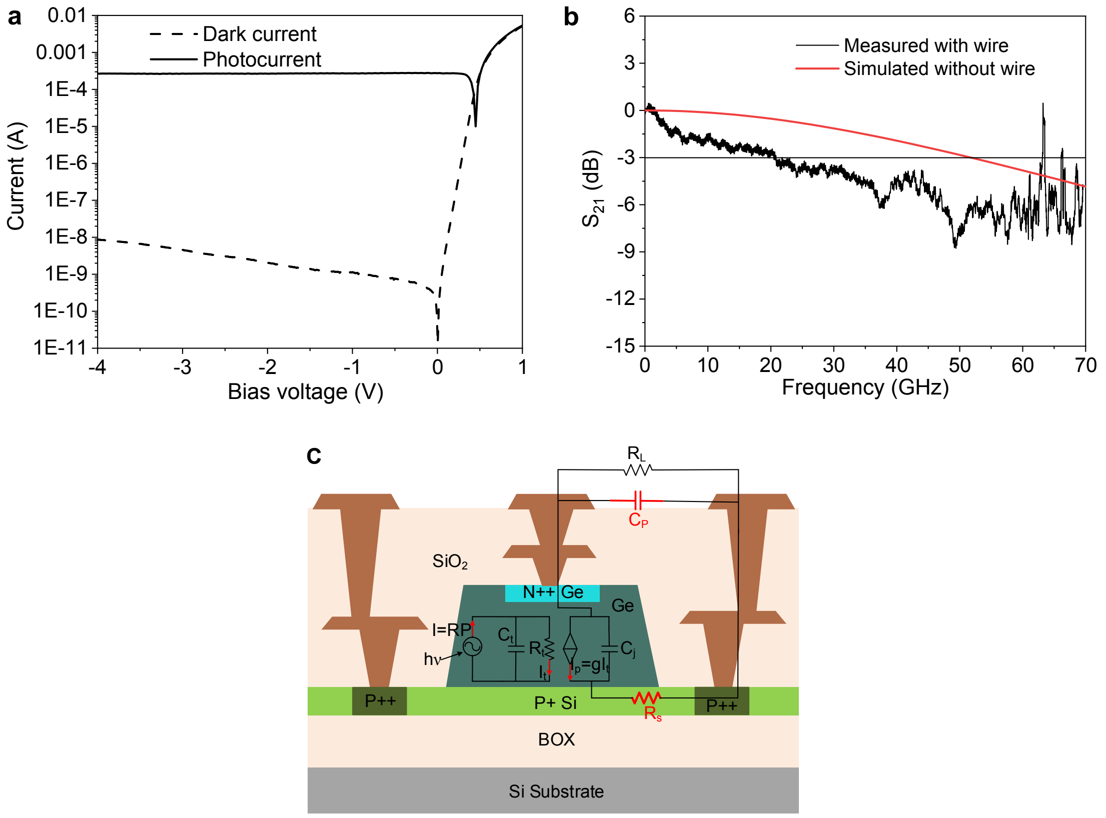

Supplementary Figure 5. Optical-to-electrical response and analysis. a The measured I-V characteristics. **b** The measured/simulated S_{21} frequency responses of the AONU with/without bonding wires. **c** The equivalent circuit model of the photodiode.

Supplementary Note 7. **Influence of doping**

As shown in Supplementary Fig. 1b, N++ Ge and P+ Si doping contribute to the formation of the n-i-p photodiode. They introduce the built-in electric field that facilitates the transport of carriers, thereby suppressing the carrier accumulation and optical nonlinearity. Therefore, we are able to achieve strong nonlinearity utilizing the partial electrodes and corresponding partial doping region. We design the electrodes and doping in the outer region where the optical field is weak and the photo-generated carrier concentration is low. The doping itself affects weakly on the optical nonlinearity but more remarkably on the speed of the optical-electrical response (the bandwidth of the photodiode). A higher concentration (denoted as N_p) will reduce the series resistance of the photodiode (R_s in Supplementary Fig. 5c), and thus the bandwidth will be improved. The simulated bandwidth versus N_p is shown as the gray line in Supplementary Fig. 6a. The other circuit components are exacted from the measured S_{21} response. When N_p exceeds 1×10^{19} cm^{-3}, the bandwidth reaches a maximum value of 50 GHz. However, when the doping concentration is high enough, the bandwidth will not increase indefinitely, as it will then be limited by other factors (such as carrier transit time). In contrast, the FCA in the P+ Si region is significantly increased, as shown in the red line of Supplementary Fig. 6a. Although the Si FCA also leads to optical nonlinearity, the light it consumed does not contribute to optical monitoring, causing additional optical loss instead. Compromisingly, the doping concentration is selected as 1×10^{19} cm^{-3}.

On the other hand, the doping depth of N++ Ge changes the depletion region depth (Ge thickness - doping depth) and thus engineers the electric field and junction capacitance (C_j), affecting the bandwidth

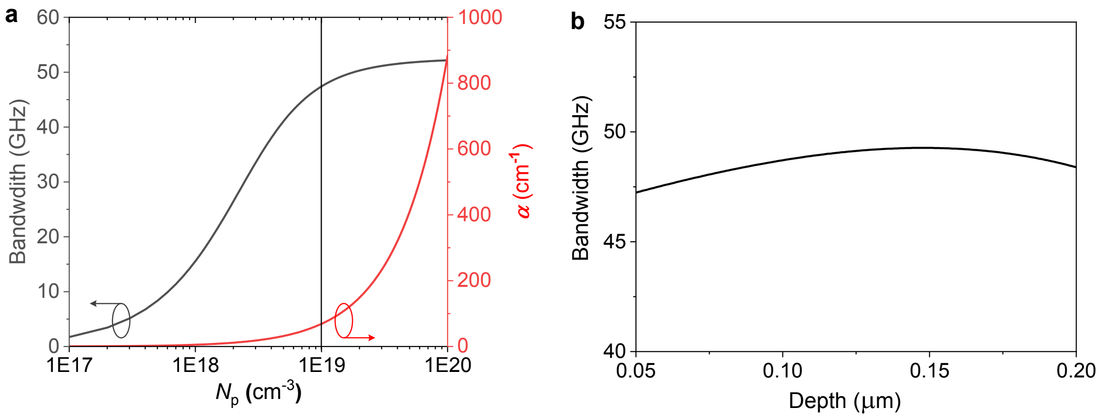

Supplementary Figure 6. Influence of doping on optical-to-electrical response. a The simulated bandwidth and FCA coefficient versus N_p. **b** The simulated bandwidth verse doping depth.

as well. The simulated bandwidth verse doping depth is shown in Supplementary Fig. 6b, showing a trend of increasing first and then decreasing. It is the result of the trade-off between carrier transit time and RC effect [4]. The simulated result shows that a doping depth of ~140 nm is optimal. However, due to the limitation of fabrication process conditions, the doping depth of N++ Ge in our device is 100 nm.

Ⅳ Neuromorphic photonic processor

Supplementary Note 8. **Training SM-AONN**

During the SM-AONN training process, the output optical power of the neuron and gradient of the weighting network are extracted through the monitoring current, when the control voltage of the weighting network changes. To understand this point, we provide the detailed method of training the SM-AONN. First, the optical monitoring is used to obtain the output optical power, for judging whether the loss function (LF) reaches the convergence threshold. Second, the optical monitoring is used to in-situ measure the gradient of each distinct weighting parameter, for updating the weighting network.

The LF is defined as the cross-entropy between forward propagation result **h** and the pre-labeled classes **y**:

$$\mathrm{LF} = \sum_{k=1}^{K}\left[-y_k \log(h_k) - (1-y_k)\log(1-h_k)\right] \tag{19}$$

where **y** is pre-known and **h** needs to be measured. Assuming that the activation function of the AONU is $P_{out}=f(P_{in})$ and the optical-electrical response is $I_{out}=g(P_{in})$, the relationship between the output optical power and the photocurrent can be obtained as $P_{out}=f[g^{-1}(I_{out})]$. Here, **h** is the P_{out} obtained from the last layer and should be deduced from the photocurrent.

We use an in-situ gradient measurement method to directly obtain the gradient of each distinct weight parameter. It is well known that the gradient for a particular weight parameter ΔW_{ij} can be obtained by computing LF(W_{ij}) and LF($W_{ij}+\delta_{ij}$), followed by the evaluation of ΔW_{ij} = (LF($W_{ij}+\delta_{ij}$)-LF(W_{ij}))/δ_{ij}. For an $N\times N$ MZI matrix, a total of $2N^2$ control voltages are required. They record the weighting values of the linear network. By giving each voltage an increment in turn, we can measure the gradient of each control voltage. When the gradient measurements are complete, the weightings can be updated using the gradient descent method. Note that the gradient measurement relies on the measurement of the LF, more fundamentally, the **h**.

From the above discussion, the neuron output optical power and gradient of the weighting network can be deduced from the photocurrent validly. However, in the saturation region of the photocurrent response (P_{in}>15 mW), the photocurrent remains almost constant (or with a small slope), and the optical power and the gradient cannot be resolved with a high resolution. As a result, the training process is terminated due to photocurrent saturation. In our implementation, we control the optical power falling in the nonlinear part of the neuron (less than 10 mW), and the photocurrent nonlinearity does not affect the training.

Supplementary Note 9. **Non-intrusive/intrusive monitoring**

We compare the performance and stability of neural networks using the proposed non-intrusive and traditional intrusive monitoring, by combining experimental and simulated results. The whole process is divided into two steps. Firstly, the optical nonlinear activation functions of the two schemes are tested experimentally. Then, the measured activation functions are used to simulate the recognition accuracy in the handwritten recognition.

The experimental set-up diagram is shown in Supplementary Fig. 7a, where we use off-chip devices combined with on-chip AONU to form the intrusive monitoring scheme. It consists of a 90:10 optical splitter, a digital-controlled variable optical attenuator (DC-VOA), a photodetector (PD) and the on-chip AONU. The electrodes of this AONU are disabled to mimic a conventional AONU. Other parameters are normalized for a fair comparison. The activation function of this scheme can be expressed as

$$P_{\text{out}} = \mathrm{f}[\left(0.9 + \delta r\right) P_{\text{in}}] \tag{20}$$

where δr is the fluctuation of the splitting ratio, and $f(\cdot)$ is the activation function of the AONU. The splitting ratio fluctuation is introduced by the DC-VOA, where the Gaussian distributed random voltages with different variances are loaded to generate a perturbed splitting ratio around 90:10. In a practical chip, this local perturbation may originate from the effective refractive index modulation of the waveguide in an optical splitter due to fabrication error, thermal crosstalk and environmental temperature fluctuation.

The measured activation functions for non-intrusive monitoring, small-perturbation and large-perturbation monitoring are shown in Supplementary Fig. 7b. We use the commonly used *tanh* function to fit the measured data and extract three different nonlinear activation functions, which are

$$\begin{aligned}
y_1 &= \max[0, \tanh(3.69 * (x - 0.075))\ ] + \mathrm{randn}(0, 0.01) \quad (\sigma_{\mathrm{r}} = 0.001) \\
y_2 &= \max[0, \tanh(3.69 * (x - 0.075))] + \mathrm{randn}(0, 0.05) \quad (\sigma_{\mathrm{r}} = 0.02) \\
y_3 &= \max[0, \tanh(3.69 * (x - 0.075))] + \mathrm{randn}(0, 0.1) \quad (\sigma_{\mathrm{r}} = 0.05)
\end{aligned} \tag{21}$$

where randn(a, b) represents a Gauss random number with mean of a and variance of b. σ_r is the variance of the extracted splitting ratio perturbation. Then, we put them into a deep neural network of scale [100, 100, 10] to simulate the accuracy. Both the first and second hidden layer covers 100 neurons and the extracted activation function is used. The third hidden layer consists of 10 neurons and the *softmax* function is used. The accuracies versus the epoch are shown in Supplementary Fig. 7c. Compared with non-intrusive monitoring, the intrusive monitoring with splitting ratio fluctuations of 0.02 and 0.05 reduces the accuracy by 1.7% and 4%, respectively. In addition, obvious accuracy fluctuations appear and this means that the system performance (accuracy) is unstable when the neural network is working. Furthermore, it can be found that the iterations for intrusive monitoring increases with the degree of perturbation to achieve an optimal accuracy, resulting in an increased training cost.

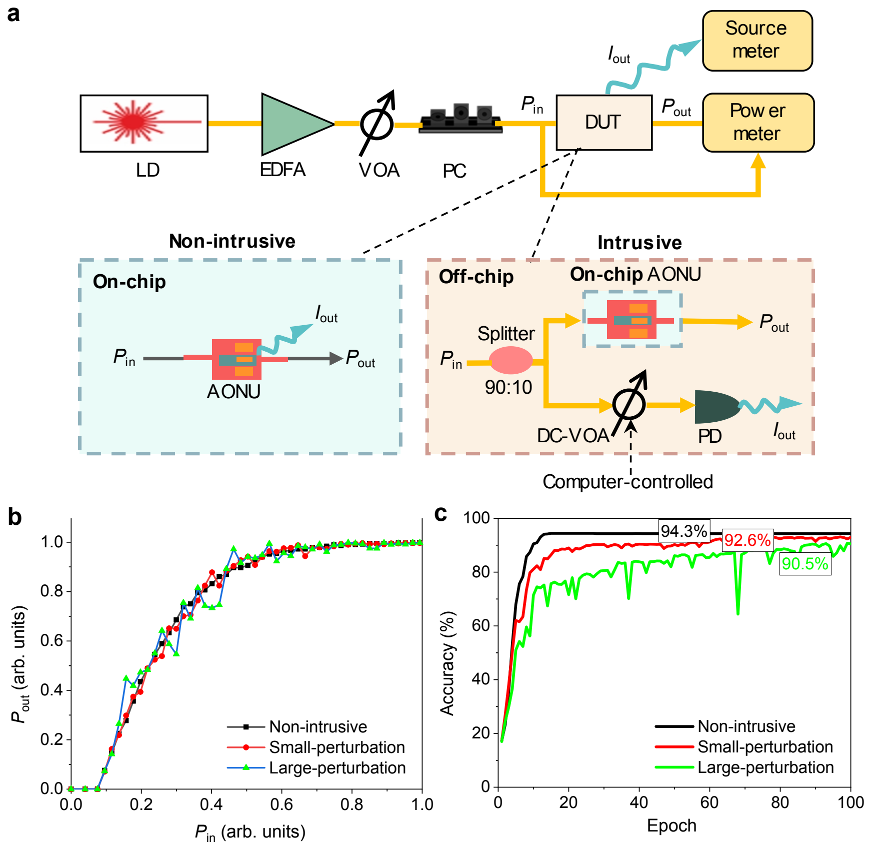

Supplementary Figure 7. Comparison between Non-intrusive/intrusive monitoring. a The experimental set-up diagram. LD, Laser. EDFA, Erbium-Doped Fiber Amplifier. VOA, Variable Optical Attenuator. PC, Polarization Controller. DUT, Device Under Test. PD, photodiode. **b** The measured activation functions for non-intrusive, small-perturbation and large-perturbation monitoring. **c** The simulated accuracies using activation functions with non-intrusive, small-perturbation and large-perturbation monitoring.

Supplementary Note 10. **Computing speed and energy**

On the device level, the speed of AONU is determined by the optical-to-optical (O/O) response and optical-to-electrical (O/E) response. Typically, the recovery time of the FCA effect is on the order of ~1 ps [5], and the time of light passing through the device is t=L/(c/n)=8 μm/(3×10^8 m/s/4.2)=0.112 ps. Thus, the total time of the O/O response of the device is about 1.1 ps, corresponding to a speed of about 0.9 THz. Compared to the ultra-fast O/O response, the speed of the O/E response is slower but is still 20.2 GHz, as shown in section 3.2. Since the proposed AONU implements both O/O response for optical nonlinearity and O/E response for optical monitoring, the maximum speed on a neuron level is up to 20 GHz (limited by the relatively slower O/E response).

The computing speed is defined as the number of operations per second (FLOPS). Assuming our system has N nodes, m layers and each layer contains an $N \times N$ weighting matrix, the system will fulfill $2mN^2$ operations once it carries out an N-dimensional matrix-vector multiplication. Due to the 20 GHz detection bandwidth, the system completes 2×10^{10} N-dimensional matrix-vector multiplications in one second. Therefore, the FLOPS of our system is

$$\text{FLOPS} = 2mN^2 \cdot 2 \cdot 10^{10} = 4m \cdot N^2 \cdot 10^{10} \quad (\text{operation/ s}) \tag{22}$$

In principle, such a computing speed is one order of magnitude faster than electronic neural networks

which are usually restricted to a GHz clock rate [6]. For our present system, m=3, N=4, and the FLOPS is 1.92×10^{12} operations per second.

Then, we estimate the power consumption of the processor. We assume the propagation loss at the circuit level is negligible because the MZI unitary matrix mesh, in principle, is lossless. Then, the power consumption mainly originates from the electrical power required to control the MZI mesh and the optical power to support the optical nonlinearities. Assuming our system has N nodes and each layer contains an $N \times N$ weighting matrix, the total number of MZI is $N(N-1)/2+N+N(N-1)/2=N^2$. The measured average power consumption for 2π phase shift per MZI is ~10 mW. Therefore, the power consumption of the MZI mesh is $10mN^2$ mW. On the other hand, according to Fig. 2e in the main text, the power consumption of each AONU is 5~10 mW to excite the optical nonlinearity. Using the maximum value of 10 mW, the total power consumption is

$$\mathrm{P} = 10m \cdot N \cdot (N+1) \ \ (\mathrm{mW}) \tag{23}$$

The energy required for computing scales with the computing speed, and the computing performance is generally evaluated by the energy consumed per operation [7]. Therefore, the energy is expressed as:

$$\mathrm{P/\,FLOPS} = 0.25 + \frac{0.25}{mN^2} \ \ (\mathrm{pJ/operation}) \tag{24}$$

In our device, P/FLOPS=0.27 pJ per operation. This power consumption is better than an "ideal" electronic computer (1 pJ per operation, assuming no energy is used on data movement) and two orders of magnitude better than conventional GPUs (100 pJ per operation) [8]. It should be pointed out that, in current configuration, the power is mainly used to maintain the working state of the MZIs, rather than optical nonlinearity. If the MZI could be set with nonvolatile phase-change materials, which would in principle require no power for maintaining, the P/FLOPS will be as low as $250/mN^2$ fJ per operation.

Supplementary References

1 Piels, M. *Si/Ge photodiodes for coherent and analog communication*. (University of California, Santa Barbara, 2013).

2 Beling, A., Xie, X. & Campbell, J. C. High-power, high-linearity photodiodes. *Optica* **3**, 328-338 (2016).

3 Shi, Y., Zhou, D., Yu, Y. & Zhang, X. 80 GHz germanium waveguide photodiode enabled by parasitic parameter engineering. *Photonics Res* **9**, 605-609 (2021).

4 Lischke, S. *et al.* Ultra-fast germanium photodiode with 3-dB bandwidth of 265 GHz. *Nat Photonics* **15**, 925-931 (2021).

5 Taghinejad, M. & Cai, W. All-optical control of light in micro-and nanophotonics. *Acs Photonics* **6**, 1082-1093 (2019).

6 Miller, D. A. Attojoule optoelectronics for low-energy information processing and communications. *J Lightwave Technol* **35**, 346-396 (2017).

7 Shen, Y. C. *et al.* Deep learning with coherent nanophotonic circuits. *Nat Photonics* **11**, 441-447 (2017).

8 Horowitz, M. in *2014 IEEE International Solid-State Circuits Conference Digest of Technical Papers (ISSCC).* 10-14 (IEEE).
